# Glaucomatous-Type Optic Discs in High Myopia

**DOI:** 10.1371/journal.pone.0138825

**Published:** 2015-10-01

**Authors:** Natsuko Nagaoka, Jost B. Jonas, Kei Morohoshi, Muka Moriyama, Noriaki Shimada, Takeshi Yoshida, Kyoko Ohno-Matsui

**Affiliations:** 1 Department of Ophthalmology and Visual Science, Tokyo Medical and Dental University, 1-5-45 Yushima, Bunkyo-ku, Tokyo, 113–8519, Japan; 2 Department of Ophthalmology, Medical Faculty Mannheim of the Ruprecht-Karls University of Heidelberg, Mannheim, 68167, Germany; Medical College of Soochow University, CHINA

## Abstract

**Purpose:**

To assess the prevalence of glaucoma in patients with high myopia defined as myopic refractive error of >-8 diopters or axial length ≥26.5 mm.

**Methods:**

The hospital-based observational study included 172 patients (336 eyes) with a mean age of 61.9±12.3 years and mean axial length of 30.1±2.3 mm (range: 24.7–39.1mm). Glaucomatous-type optic discs were defined by glaucomatous optic disc appearance. Glaucoma was defined by glaucomatous optic disc appearance and glaucomatous Goldmann visual field defects not corresponding with myopic macular changes.

**Results:**

Larger disc area (mean: 3.18±1.94 mm^2^) was associated with longer axial length (*P*<0.001; standardized correlation coefficient: 0.45). Glaucoma was detected in 94 (28%; 95% Confidence intervals: 23%, 33%) eyes. In multivariate analysis, glaucoma prevalence was 3.2 times higher (*P*<0.001) in megalodiscs (>3.79 mm^2^) than in normal-sized discs or small discs (<1.51 mm^2^) after adjusting for older age. Axial length was not significantly (*P* = 0.38) associated with glaucoma prevalence in that model. Glaucoma prevalence increased by a factor of 1.39 for each increase in optic disc area by one mm^2^. Again, axial length was not significantly (*P* = 0.38) associated with glaucoma prevalence when added to this multivariate model.

**Conclusion:**

Within highly myopic individuals, glaucoma prevalence increased with larger optic disc size beyond a disc area of 3.8 mm^2^. Highly myopic megalodiscs as compared to normal sized discs or small discs had a 3.2 times higher risk for glaucomatous optic nerve neuropathy. The increased glaucoma prevalence in axial high myopia was primarily associated with axial myopia associated disc enlargement and not with axial elongation itself.

## Introductions

Previous hospital-based studies and population-based investigations have shown that myopia, in particular high axial myopia, can be a risk factor for glaucomatous optic neuropathy [1–14]. It has remained unclear, which factors associated with myopia were responsible for the increased susceptibility for glaucomatous optic nerve damage in myopic eyes. Histological studies reported on morphological particularities in eyes with axial high myopia. These features included a thinning and stretching of the lamina cribrosa in the highly myopic secondary macrodiscs (also called megalodiscs), and an elongation and thinning of the peripapillary scleral flange in the parapapillary region of highly myopic optic nerve heads [15–20]. Since most of the previous clinical studies did not clearly differentiate between high myopia (defined as an axial length of more than 26.5 mm or a myopic refractive error of more than -8 diopters) and moderate myopia with a lower degree of axial elongation, and since population-based studies usually did not include highly myopic individuals in a number sufficient for a powerful statistical analysis, we carried out this study and examined a relatively large group of highly myopic individuals who attended a clinics specialized on high myopia.

## Methods

The hospital-based observational retrospective clinical study included patients with high myopia who had consecutively been examined in the High Myopia Clinic of the Tokyo Medical and Dental University between January 2012 and December 2012, and for whom optic disc photographs were available. The study was approved by the Ethics Committee of the Tokyo Medical and Dental University and followed the tenets of the Declaration of Helsinki. Due to the retrospective analysis of data which had already been obtained in routine clinical taking care of patients, the ethics committee decided that an informed consent signed by the individual patients was not necessary. Patient records/information was anonymized and de-identified prior to analysis. High myopia was defined as a myopic refractive error of more than -8 diopters or an axial length ≥26.5 mm.

All patients underwent a comprehensive ophthalmological examination including refractometry and measurement of best corrected visual acuity, biometry (IOL Master; Carl Zeiss Meditec Co., Jena, Germany), Goldmann applanation tonometry, gonioscopy, and 50 degree color fundus photography (TRC-50DX, Topcon, Tokyo, Japan). We measured the area of optic disc on the fundus photographs by using the software of the imaging system (PDT/MPS software; Topcon, Tokyo, Japan) and by taking into account axial length for correction of the fundus image magnification by the optic media of the eye. Based on the findings of the Beijing Eye Study, the optic discs were classified into three types: “Small discs” were defined as discs smaller than 1.51 mm^2^, and "megalodiscs" as discs larger than 3.79 mm^2^. “Normal sized discs” were the remaining ones [10].

All optic disc photographs were additionally examined for a glaucomatous-type optic disc appearance by a glaucoma specialist (JBJ). We differentiated between absolute criteria and relative criteria for the diagnosis of a glaucomatous-type optic disc. The list of absolute criteria included a notch in the neuroretinal rim in the temporal inferior disc region or the temporal superior disc region, or localized retinal nerve fiber layer defects which could not be explained by any other cause than glaucoma, or an abnormally large cup size as compared with the optic disc size. Relative criteria were a neuroretinal rim being markedly thinner in the inferior disc region than in the superior disc region, even if the smallest neuroretinal rim part was located in the temporal horizontal disc region; a diffuse decrease in the visibility of the retinal nerve fiber layer (particularly in eyes with small discs), if there were no other reasons than glaucoma for a retinal nerve fiber layer loss; and an optic disc hemorrhage, if there were no other causes for disc hemorrhages. If none of the absolute criteria were positive, at least 2 relative criteria had to be fulfilled. All photographs were examined in a masked manner by two experienced examiners (NN, JBJ) independently of each other. At the time of the evaluation, the examiners were not aware of the perimetric findings nor other clinical data such as intraocular pressure.

Goldmann perimetry was carried out by one of two trained perimetrists who had been performing Goldmann perimetry as their main professional activity for more than 20 years. A glaucomatous visual field defect was defined as a nasal step defect respecting the horizontal meridian, a temporal wedge-like defect or an arcuate defect often extending from the blind spot, a paracentral defect 10–20° from the blind spot located in the Bjerrum region and which did not correspond to a myopic lesion in the macular region, an arcuate defect with peripheral breakthrough, and a generalized constriction of the visual field. Perimetric defects in the visual center or other defects located in the central visual field and which corresponded with myopic macular changes were not considered. Since the visual field was tested using a kinetic Goldmann perimetry, quantitative indices on the reliability of the test results, such as number of fixation losses and rate of false negative results or false positive results were not obtained. However, any unreliability of the test results would have been mitigated by the experienced technicians.

We recorded the prevalence of glaucomatous-type optic discs if the criteria mentioned above were fulfilled, and we recorded the prevalence of glaucoma, defined as the presence of a glaucomatous optic disc appearance and of glaucomatous visual field defects.

For the statistical analysis, we used a commercially available statistical software program (SPSS version 22.0; IBM-SPSS, Chicago, IL, USA). Parameters were described by their means and standard deviations. We carried out a binary regression analyses to assess relationships between the prevalence of glaucomatous-type discs or the prevalence of glaucoma and other parameters. Eventually, we performed a multivariate analysis in which the prevalence of glaucomatous-type discs or the prevalence of glaucoma was the dependent variable and all parameters, which were significantly associated with the prevalence of glaucomatous-type discs or the prevalence of glaucoma in the univariate analysis, were included into the list of independent variables. Odds ratios (OR) and 95% confidence intervals (CI) were assessed. A *P*-value of less than 0.05 was considered to be statistically significant.

## Results

The study included 336 eyes of 172 patients (119 women). Six of the 172 patients had unilateral high myopia, one eye of a patient with bilateral high myopia was excluded because of a history of vitreoretinal surgery, and one eye of another patient with bilateral high myopia was excluded because of a history of optic neuritis which could have affected the visual fields. Mean age was 61.9 ± 12.3 years (range; 30 to 89 years), and mean axial length was 30.1 ± 2.3 mm (range; 24.7 to 39.1 mm). In all eyes, the anterior chamber angles were widely open upon gonioscopy. The mean intraocular pressure measured 14.3 ± 2.8 mmHg (range: 7 to 24 mmHg). Intraocular pressure was lower than 21 mmHg in 334 (99.4%) of the 336 eyes.

**Fig 1:**
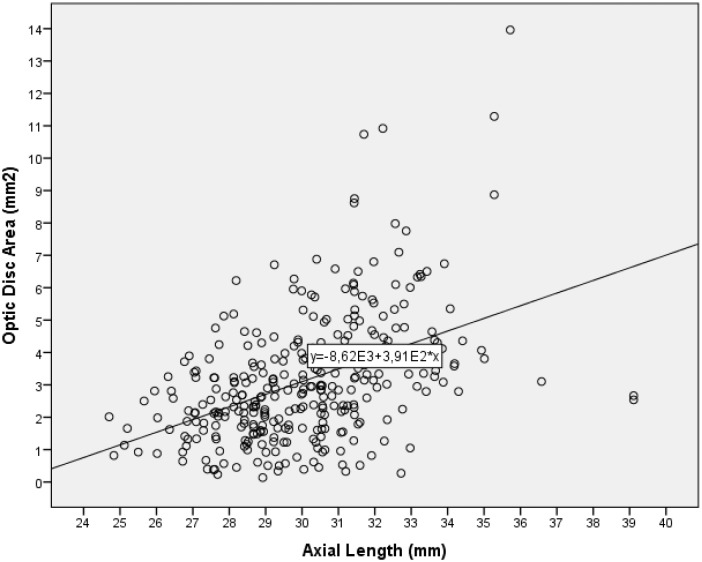
Correlation between axial length and optic disc size. In univariate analysis, optic disc area was significantly associated with axial length (*P*<0.001).

Mean optic disc area was 3.18 ± 1.94 mm^2^ (median: 2.81 mm^2^). In univariate analysis, larger optic disc area was significantly associated with longer axial length (*P*<0.001; standardized correlation coefficient r: 0.45; equation of the regression line: Optic Disc Area (mm^2^) = 0.39 x Axial Length (mm)– 8.62) (Fig 1) and with younger age (*P*<0.001; r: -0.32).

**Fig 2:**
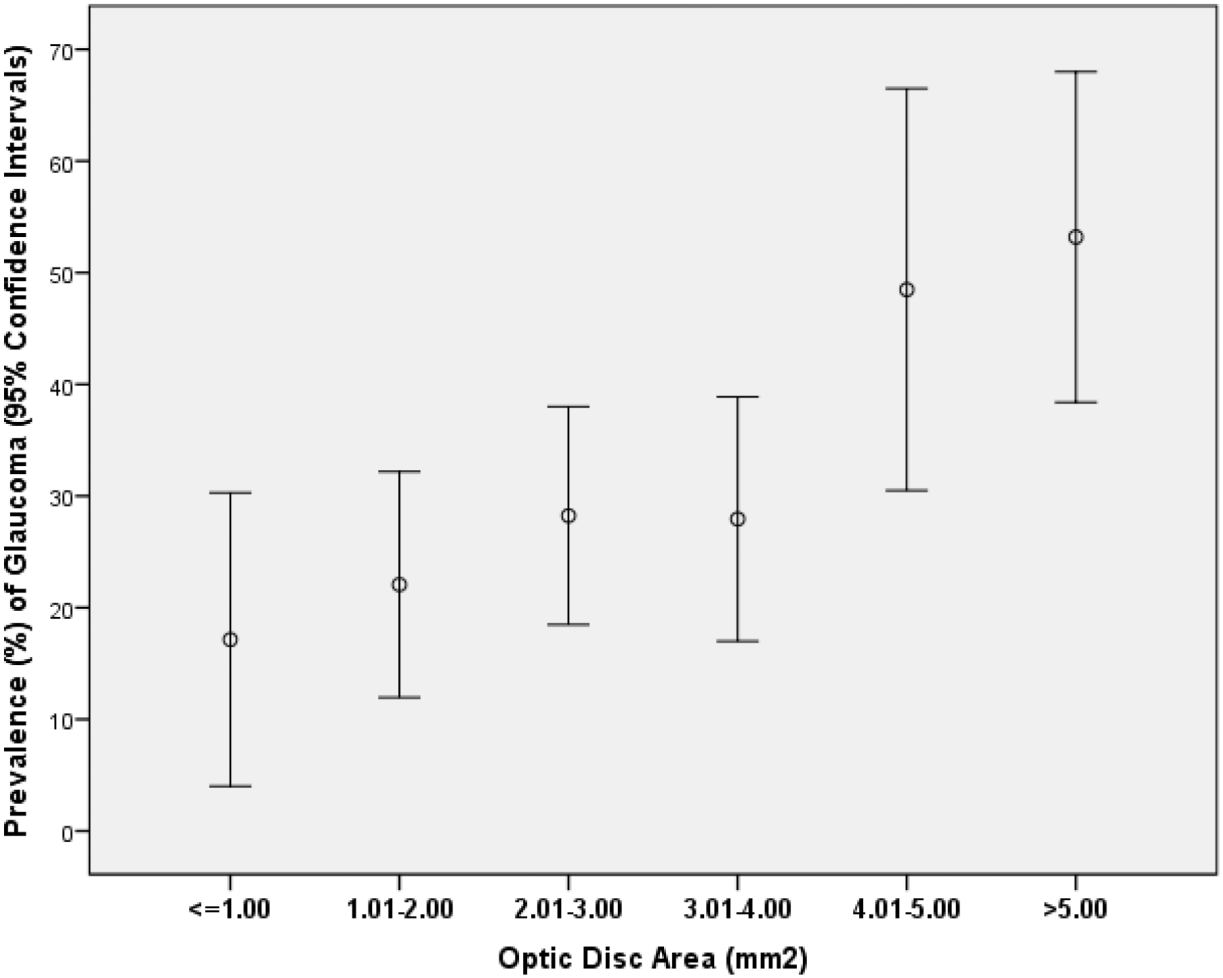
Prevalence of glaucomatous optic nerve damage in highly myopic individuals stratified by optic disc size. In univariate analysis, the prevalence of glaucomatous-type discs increased significantly with optic disc area (*P*<0.001).

Out of the 336 eyes included into the study, 105 (31.3%; 95% CI: 26%, 36%) eyes showed a glaucomatous-type optic disc (Table 1). Both examiners had disagreed in the assessment of two optic discs, which were then together assessed and discussed. The prevalence of glaucomatous-type discs did not differ significantly (*P* = 0.73) between the group of small discs (14/64 or 21.9%; 95% CI: 11%, 32%) and the group of normal-sized discs (42/173 or 24.3%, 95%CI: 18%, 31%) while it was significantly (*P*<0.001) higher in the group of megalodiscs (49/99 or 49.5%, 95% CI: 39%, 60%). In univariate analysis, the prevalence of glaucomatous-type discs increased significantly with optic disc area (*P*<0.001; OR: 1.29; 95%CI: 1.14, 1.47) (Fig 2), older age (*P* = 0.039; OR: 1.02; 95%CI: 1.001, 1.04), and with longer axial length (*P*<0.001; OR: 1.18; 95%CI: 1.06, 1.31). It was positively associated with taken anti-glaucomatous medication (*P*<0.001). The prevalence of glaucomatous-type discs was not significantly associated with gender (*P* = 0.49),

**Table 1:** Characteristics (Mean ± Standard Deviation; Range) of the Study Population.

	Glaucomatous Type of Optic Disc	Glaucomatous Optic Disc and Visual Field Defects
Number of Eyes	105	94
Age (Years)	63.7 ± 11.9 (39–87)	64 ± 12.1 (39–87)
Refractive Error (Diopters)	-14.1 ± 5.4 (-4 to -22.5)*	-14.3 ± 5.2 (-4 to -22.5)*
Axial Length (mm)	30.6 ± 2.2 (25.5–35.7)	30.6 ± 2.2 (25.5–35.7)
Intraocular Pressure-Lowering Medication (Eyes)	76	73

* High myopia was defined as a myopic refractive error of more than -8 diopters or an axial length ≥26.5 mm. All eyes with a myopic refractive error of less than -8 diopters had an axial length of ≥26.5 mm. For the calculation of the refractive error, pseudophakic eyes and aphakic eyes were excluded

In multivariate regression analysis, higher prevalence of glaucomatous-type optic discs was associated with older age (*P* = 0.002) and presence of a megalodiscs (*P*<0.001) (Table 2). In that model, patients with megalodiscs were about four times more likely to have glaucomatous optic discs as compared with individuals with normal-sized discs or individuals with small discs. Results of the associations of glaucomatous-type optic discs with various factors remained similar when the eyes with small discs (defined by an optic disc area of less than 1.51 mm2) were excluded from the analysis (data not shown). Higher prevalence of glaucomatous-type optic discs was associated with older age (*P* = 0.03; B: 1.04; 95%CI: 1.003, 1.065) and presence of a megalodiscs (*P*<0.001; B: 4.47; 95%CI: 2.14, 9.37), so that eyes with megalodiscs had about an 4.5 times higher probability to have a glaucomatous optic discs as compared with eyes with normal-sized discs. If taking anti-glaucomatous medication was added to the list of independent variables, it was significantly associated with higher prevalence of glaucomatous-type optic discs (*P*<0.001; B: 3.26; 95%CI: 1.93, 5.51) after adjusting for older age (K = 0.005) and presence of a megalodisc (*P*<0.001). Since however, taking medication is the consequence of a glaucomatous appearance of the optic disc (otherwise, there were no major reason to take anti-glaucomatous medication), and since the analysis was aimed at causative factors for the prevalence of glaucomatous-type optic nerve heads, we dropped taking medication from the further statistical analysis. Axial length was not significantly (*P* = 0.19) associated with the prevalence of glaucomatous-type optic discs in that model. If optic disc size group was replaced by axial length in the multivariate analysis, prevalence of glaucomatous-type optic discs was associated with older age (*P* = 0.04; OR: 1.02; 95%CI: 1.001, 1.04) and longer axial length (*P* = 0.002; OR: 1.18; 95%CI: 1.06, 1.32). If only one randomly selected eye per individual was included into the statistical analysis, higher prevalence of glaucomatous-type optic discs was associated with older age (*P* = 0.03; B: 1.03; 95%CI: 1.003, 1.065) and presence of a megalodiscs (*P*<0.001; B: 4.74; 95%CI: 2.14, 9.37). Again, axial length was not significantly (*P* = 0.08) associated with the prevalence of glaucomatous-type optic discs in that model. If optic disc size group was replaced by axial length in the multivariate analysis, prevalence of glaucomatous-type optic discs was associated with longer axial length (*P* = 0.002; OR: 1.29; 95%CI: 1.10, 1.51), but not with age (*P* = 0.09).

**Table 2:** Associations (multivariate analysis) between the prevalence of glaucomatous optic disc appearance or glaucoma (defined by glaucomatous optic disc appearance and glaucomatous visual field defects) as dependent variable and age and presence of a megalodisc as independent variables.

Parameter	*P*-Value	Odds Ratio	95% Confidence Interval
Glaucomatous Optic Disc Appearance			
Age (Years)	0.002	1.03	1.01, 1.06
Megalodisc (No/Yes)	<0.001	3.96	2.32, 6.74
Glaucoma (Defined by Glaucomatous Optic Disc Appearance and Glaucomatous Visual Field Defects)			
Age (Years)	0.002	1.04	1.01, 1.06
Megalodisc (No/Yes)	<0.001	3.23	1.83, 5.69

In a similar manner, if optic disc size instead of the presence of megalodiscs was included into the list of independent parameters, prevalence of glaucomatous-type discs increased significantly with enlarging optic disc size after adjusting for older age (Table 3). In this model, axial length was again not significantly (*P* = 0.40) associated with glaucomatous-type discs prevalence when added to the multivariate model. If only one randomly selected eye per individual was included into the statistical analysis, prevalence of glaucomatous-type discs increased significantly with enlarging optic disc size (*P*<0.001; B: 1.46; 95%CI: 1.20, 1.77) after adjusting for older age (*P* = 0.007; B: 1.05; 95%CI: 1.01, 1.08). In this model, axial length was again not significantly (*P* = 0.13) associated with glaucomatous-type discs prevalence when added to the multivariate model.

**Table 3:** Associations between the prevalence of glaucomatous optic neuropathy as dependent variable and age and optic disc area as independent variables.

Parameter	*P*-Value	Odds Ratio	95% Confidence Interval
Glaucomatous Optic Disc Appearance			
Age (Years)	<0.001	1.04	1.02, 1.07
Optic Disc Size (mm^2^)	<0.001	1.42	1.24, 1.63
Glaucoma (Defined by Glaucomatous Optic Disc Appearance and Glaucomatous Visual Field Defects)			
Age (Years)	<0.001	1.05	1.02, 1.07
Optic Disc Size (mm^2^)	<0.001	1.39	1.19, 1.64

If glaucoma was defined by a glaucomatous optic disc appearance and glaucomatous visual field defects, 94 (28%) eyes fulfilled the definition. Mean disc area was 3.73 ± 2.18 mm^2^. The prevalence of glaucoma did not differ significantly (*P* = 0.60) between the group of small discs (12/64 or 18.8%; 95% CI: 9%, 29%) and the group of normal-sized discs (39/173 or 22.5%, 95%CI: 16%, 29%) while it was significantly (*P*<0.001) higher in the group of megalodiscs (43/99 or 43.4%, 95% CI: 34%, 53%). In univariate analysis, the prevalence of glaucoma increased significantly with optic disc area (*P* = 0.002; OR: 1.26; 95%CI: 1.09, 1.46), older age (*P* = 0.02; OR: 1.03; 95%CI: 1.01, 1.06), and with longer axial length (*P* = 0.02; OR: 1.15; 95%CI: 1.02, 1.29). In multivariate regression analysis, higher prevalence of glaucomatous optic discs was associated with older age (*P* = 0.002) and presence of a megalodiscs (*P*<0.001) (Table 2). In that model, patients with megalodiscs were 3.2 times more likely to have glaucomatous optic discs as compared with individuals with normal-sized discs or individuals with small discs. Axial length was not significantly (*P* = 0.38) associated with the prevalence of glaucomatous optic discs in that model. If optic disc size group was replaced by axial length in the multivariate analysis, prevalence of glaucomatous optic discs was associated with older age (*P* = 0.04; OR: 1.03; 95%CI: 1.001, 1.05) and longer axial length (*P* = 0.03; OR: 1.15; 95%CI: 1.02, 1.29). In a similar manner, if optic disc size instead of the presence of megalodiscs was included into the list of independent parameters, prevalence of glaucoma increased significantly with enlarging optic disc size after adjusting for older age (Table 3). In this model, axial length was again not significantly (*P* = 0.67) associated with glaucoma prevalence when added to the multivariate model. If only one randomly selected eye per individual was included into the statistical analysis, higher prevalence of glaucomatous optic discs was associated with older age (*P* = 0.04; B: 1.03; 95%CI: 1.002, 1.064) and presence of a megalodiscs (*P*<0.001; B: 3.88; 95%CI: 1.84, 8.19). Axial length was not significantly (*P* = 0.13) associated with the prevalence of glaucomatous-type optic discs in that model. If optic disc size group was replaced by axial length in the multivariate analysis, prevalence of glaucomatous-type optic discs was associated with longer axial length (*P* = 0.006; OR: 1.25; 95%CI: 1.07, 1.47), while was not significantly associated with age (*P* = 0.10). If optic disc size instead of the presence of megalodiscs was included into the list of independent parameters, prevalence of glaucoma increased significantly with enlarging optic disc size (*P* = 0.001; B: 1.41; 95%CI: 1.16, 1.70) after adjusting for older age (*P* = 0.01; B: 1.04; 95%CI: 1.01, 1.08). In this model, axial length was again not significantly (*P* = 0.19) associated with glaucoma prevalence when added to the multivariate model.

## Discussion

In our hospital-based study of highly myopic patients, the prevalence of glaucomatous-type discs was 31%, and the prevalence of glaucoma as defined by a glaucomatous appearance of the optic nerve head and visual field defects was 28%. The prevalences of both parameters were significantly (*P*<0.001) higher in megalodiscs (50% and 43%, respectively) than in normal-sized discs (24% and 23%, resp.) or in small discs (22% and 19%, resp.). After adjusting for age, the frequency of glaucomatous-type discs risk and the frequency of glaucoma were about four times higher and 3.2 times higher, resp., in the megalodisc group than in the remaining eyes. If optic disc size instead of the presence of megalodiscs was included into the list of independent parameters, prevalence of glaucomatous-type discs increased by a factor of 1.42 and by a factor of 1.39, resp., for each increase in optic disc area by one square millimeter (Table 3) (Fig 2). As a corollary, prevalence of glaucomatous-type discs and prevalence of glaucoma increased significantly by 18% and by 15%, resp., with each mm increase in axial length after adjusting for age.

The results of our study agree with the findings obtained in preceding hospital-based studies and in epidemiologic studies which have also suggested an association between glaucomatous optic nerve damage and moderate to high myopia [1–12]. In the Barbados Eye Study on black individuals, myopia was associated with higher odds of having glaucoma while hyperopia was associated with lower odds of glaucoma [1]. In the Blue Mountains Eye Study, the prevalence of glaucoma was higher individuals with moderate to high myopia (4.4%) than in emmetropic individuals (1.5%) [3]. The Malmö Eye Survey examined more than 30,000 individuals in whom the prevalence of glaucoma increased with increasing myopia. In that study, the association of myopia with glaucoma was stronger at lower intraocular pressure levels than at higher intraocular pressure levels [21]. The findings of our study are also in agreement with the results of the population-based Beijing Eye Study in which a higher prevalence of glaucomatous optic neuropathy was associated with a myopic refractive error exceeding -8 diopters [10].

Our study extended the results of the preceding studies by the finding that in eyes with high axial myopia a large optic disc size as compared with a long axial length was more important for the association with an increased prevalence of glaucomatous-type optic nerve heads or increased prevalence of glaucoma. In the multivariate analysis, the prevalence of glaucomatous-type optic discs and the glaucoma prevalence increased with larger disc size (or the presence of a megalodisc) after adjusting for older age, while in the same model axial length was not significantly associated with the prevalence of glaucomatous-type optic discs or with the prevalence of glaucoma. If disc size was dropped from the model, longer axial length became significantly associated with higher prevalence of glaucomatous-type optic discs and with higher glaucoma prevalence. The correlation coefficients were however lower than those for the disc size parameter. One may infer that the axial myopia related enlargement of the optic nerve head including the elongation and thinning of the parapapillary scleral tissue may be the main factor leading to the association between high axial myopia and an increased prevalence of glaucomatous-type optic discs.

One of the reasons for the increased glaucoma susceptibility in highly myopic eyes with secondary macrodiscs (or megalodiscs) may be histological changes in the lamina cribrosa and in the peripapillary tissue [15, 16]. Previous histomorphometric investigations revealed that eyes with myopic axial elongation as compared to eyes with normal axial length showed a marked thinning of the lamina cribrosa [15]. It has been postulated that the thinning of the lamina cribrosa led to shortening of the distance between the intraocular compartment and the retrobulbar optic nerve compartment. A shortened distance between both compartments leads to a steepening of the pressure gradient across the lamina cribrosa [21]. This situation may be comparable to the one found in eyes with advanced glaucoma, which is characterized by a thinning of the lamina cribrosa and an increased susceptibility for further progression of the disease [22, 23]. Another morphologic reason for the increased glaucoma prevalence in high myopia may be changes in the parapapillary region. In eyes with normal axial length, the peripapillary scleral flange has a length of about 0.5 mm and a thickness of about 0.5 mm [16]. The peripapillary scleral flange is the continuation of the inner half of the posterior sclera and continues into the lamina cribrosa. In eyes with an axial length exceeding 26.5 mm, the peripapillary scleral flange gets markedly elongated and thinned. It is associated with the presence of a parapapillary gamma zone and parapapillary delta zone [16–18]. In some highly myopic eyes, the length of the peripapillary scleral flange may be as long as 5 mm while its thickness may be reduced to as little as 50 μm. Since the peripapillary scleral flange is the biomechanical anchor of the lamina cribrosa, the high axial myopia associated elongation and thinning of the peripapillary scleral flange may have consequences for any process in the lamina cribrosa including the susceptibility for glaucomatous optic neuropathy.

Our study has potential limitations. First, our study was focused on the prevalence of glaucoma in highly myopic eyes. Diagnosis of glaucoma in highly myopic eyes is however, difficult due to several reasons including primary myopic changes in the morphology of the optic nerve head and retinal nerve layer appearance, frequent non-glaucomatous reasons for visual field defects, and unreliable results of digitized imaging techniques. In our study, we used however two definitions or parameters, the prevalence of a glaucoma-like appearance of the optic nerve head and the prevalence of glaucoma as defined by the occurrence of a glaucoma-like optic nerve head appearance and by the presence of glaucomatous visual field defects. Since visual field defects in axially highly myopic eyes can be due to myopic changes in the macular region, we defined glaucomatous visual field defects as lesions which did not correspond to myopic macular changes and the shape and location of which were typically glaucomatous such as arcuate defects or nasal steps. Second, inclusion criterion for our study population was a myopic refractive error of more than -8 diopters or an axial length ≥26.5 mm. All findings obtained in our study were therefore valid only for the highly myopic group of individuals. Third, as in any hospital-based study, a bias due to a referral selection of the patients was possible. It may have been unlikely however, that highly myopic patients attended the High Myopia Clinics because of a particular size of their optic nerve heads. It may have also been unlikely, that the study participants attended the High Myopia Clinics because they had glaucoma since the High Myopia clinics has been organized and led by retina specialists. The potential disadvantage of a study design with hospital-based patient recruitment was combined with the advantage of a relatively high number of individuals affected with high myopia. Based on the study population of the Beijing Eye Study in which about 1.6% of the study participants had an axial length of >26.5 mm, one would have needed a study sample of 11,466 participants to arrive at a similar number of highly myopic individuals in a population-based investigation as we had in our hospital-based study [10, 20]. Fourth, the optic disc was measured on two-dimensional fundus photographs. Since the optic disc, in particular in highly myopic eyes, is not located at the posterior fundus pole, the perspective view onto the optic disc gets perspectively distorted. This perspective distortion of the image of the optic disc leads to falsely low measurements of the disc diameter which is orientated perpendicularly to the axis of the disc rotation. One can differentiate between a disc rotation around the vertical axis what leads to a perspective shortening of the horizontal disc diameter; a disc rotation around the horizontal axis, what leads to a perspective shortening of the vertical disc diameter; and a disc rotation around the sagittal axis, which does not change the perspective view on the optic nerve head and thus does not influence the two-dimensional assessment of the disc diameters. Future studies may use optic nerve head images obtained by optical coherence tomography which allows a three-dimensional assessment of the optic disc rotations and of the real disc diameters independently of a perspective distortion [24]. Fifth, the detection of a glaucomatous optic nerve appearance is generally easier in large optic discs than in small discs, if the small discs do not have cupping. One may therefore argue that the lower prevalence of detected glaucomatous-type optic discs in the group of eyes with small discs than in the group of eyes with megalodiscs may have been due to a diagnostic bias. Interestingly, however, the frequency of glaucomatous-type discs did not differ between the group of eyes with small optic discs and the group of eyes with medium sized optic discs. It may thus have been unlikely that a diagnostic bias was responsible for the association between higher prevalence of glaucomatous-type discs and larger disc size beyond a disc area of about 3.7 mm^2^. In a similar manner, one may argue that the group of megalodiscs was prone to an overdiagnosis of glaucomatous-type discs since large discs already physiologically have large optic cups [25]. The examiners however were aware of that potential bias [25]. Sixth, stratifying the whole glaucoma group by the severity of the disease might have been helpful to further explore the association between glaucomatous optic nerve damage and disc size in the highly myopic eyes. Since however most of the eyes showed signs of myopic retinopathy leading to visual field defects in addition to the glaucomatous perimetric defects, the amount of visual field defect was not sufficiently specific for glaucomatous optic nerve damage. Also, precise measurements of the neuroretinal rim was difficult due to the decreased spatial contrast and color contrast between the neuroretinal rim and the optic cup in the highly myopic eyes. In addition, the neuroretinal rim size may depend on the disc size also in highly myopic eyes as it does in non-highly myopic eyes [25]. This relationship has however not been fully explored in non-glaucomatous discs of highly myopic individuals yet, so that no normative values have been available to set into relationship the neuroretinal rim area with disc size in the glaucomatous highly myopic eyes.

In conclusion, the prevalence of glaucomatous-type optic discs and the prevalence of glaucoma in highly myopic individuals increased with larger optic disc size after adjusting for age. Highly myopic megalodiscs as compared to normal sized discs or small discs had a 4 times higher risk for glaucomatous-type discs and of glaucoma. The increased prevalence of glaucomatous-type discs and of glaucoma in axial high myopia was primarily associated with the axial myopia associated optic disc enlargement, and not with axial elongation itself.
